# Body height among adult male and female Swiss Health Survey participants in 2017: Trends by birth years and associations with self-reported health status and life satisfaction

**DOI:** 10.1016/j.pmedr.2022.101980

**Published:** 2022-09-12

**Authors:** Sarah-Maria Müller, Joël Floris, Sabine Rohrmann, Kaspar Staub, Katarina L Matthes

**Affiliations:** a Institute of Evolutionary Medicine, University of Zurich, Switzerland; b Epidemiology, Biostatistics, and Prevention Institute, University of Zurich, Switzerland; c Swiss School of Public Health (SSPH+), Zurich, Switzerland; d Department of History, University of Zurich, Switzerland

**Keywords:** Stature, Trend, Health

## Abstract

The increase in adult height for 150 years is linked to overall improvements in nutrition, hygiene, and living standards. Height is positively associated with general health and success on various levels (e.g. quality of life, earnings or happiness). The aim of this study was to investigate whether different subgroups show different trends across birth cohorts. We wanted to know whether taller individuals considered themselves as healthier and their quality of life as better than shorter individuals.

We included 19,435 participants from the Swiss population-based Health Survey 2017. GAM were used to assess nonlinear associations between height and birth year. Multinomial logistic regression was used to predict probabilities of self-rated health in relation to height.

The increase in average height slows down from the 1970s birth cohorts. Participants with parents from Central/Northern/Western Europe (men 177.9 cm, women: 165.1 cm) or Eastern Europe (men 178.7 cm, women: 165.7 cm) were taller than participants with parents from South America (men 174.3 cm, women: 161. cm) and Asia (men 173.2 cm, women: 160.1 cm). Participants with tertiary education were taller than participants from education levels (mean difference men: 4.5 cm, women: 5.0 cm). Height was positively associated with self-declared aspects of health and life satisfaction.

These results support the conclusion that body height as a co-factor of health aspects should be considered in public health research. Although adult body height can no longer be influenced, nutritional status and thus also healthy growth can be influenced in childhood by public health programs, by eliminating social inequalities, and by strengthen healthy living conditions.

## Highlights

• Increase in average body height had slowed down from around the 1970s birth cohorts.
• Women and men with tertiary education levels were taller than participants holding other education levels.
• Taller participants were less overweighted and were more satisfied with their lives than shorter participants.
• Taller participants had better overall health than shorter participants.
• Taller men were more likely to have lower back pain than shorter men.

## 1 Introduction

The determinants of growth and adult height include both genetics and environmental factors such as nutrition, socioeconomic status, and health, and their complex interaction results in phenotypic variation (Stulp and Barrett, 2016). A recent major global study showed that the difference in mean body height between the tallest and shortest populations on Earth is around 20 cm (Bentham et al., 2016). At the individual level, genetics is the most important influencing factor. Genes provide the roadmap and developmental plan for growth; they set a framework: the genetic growth potential (McEvoy and Visscher, 2009). Following twin studies within affluent populations, the heritability of body height is about 80 percent (Visscher et al., 2010). For the remaining about 20 percent and within the same population, environmental factors then determine the exact direction of development within the framework set by the genes. Looking at the average height of a population or subgroups gives access to the biological or health side of the standard of living (Floris et al., 2019). Variations in average body height over time and between subpopulations are attributed to differences in living conditions, health and well-being (McEvoy and Visscher, 2009, Steckel, 2009, Cole, 2003, Komlos, 2010, Deaton, 2007). Previous studies suggest that the increase in adult height occurring in developed countries over the past two centuries is linked to overall improvements in nutrition, hygiene/sanitation, and living standards and decreasing exposure to infectious diseases (Perkins et al., 2016).

In the 1970s, the average body height of populations in Central and Northern Europe stopped growing and reached a plateau (Vinci et al., 2019). Currently, reasons such as a reached genetic upper limit, increasing social inequality, or the ethnic composition of these populations are listed as explanatory factors for this phenomenon (Rohrmann et al., 2017). Therefore, monitoring not only growth in children but also adult height is beneficial, as it may be used as a potential marker to make statements about a population’s environment, including socioeconomic status, inequality, nutrition, and overall health status (Perkins et al., 2016). There has been very little research on recent monitored trends in body height in connection to socioeconomic and/or migration background within a given population. As one of the few examples, a study conducted in the Netherlands has shown that the increase in secular height in Dutch children came to a halt, while a positive trend in body height in both Turkish and Moroccan children living in the Netherlands remained (Schönbeck et al., 2015).

Within the populations of modern Westernized countries, height is associated with consequences for an individual’s health and success on various levels (e.g. health, happiness, earnings or reproduction) (Perkins et al., 2016). Being tall is linked with greater career chances and higher salaries (Herpin, 2005, Magnusson et al., 2006, Gautschi and Hangartner, 2006, Heineck, 2005, Hübler, 2009, Height, 2019). In terms of morbidity and mortality (Engeland et al., 2003) being taller is negatively associated with cardiovascular diseases but positively associated with certain types of cancer or musculoskeletal health (Height, 2019, The Emerging Risk Factors Collaboration, 2012). This could also be related to the fact that taller people are less likely to be overweight on average (Rickenbacher et al., 2022). Height has been shown to be associated with better ratings of life satisfaction (Baten, 2017). On average, taller people assess their lives as more favourable, as they are more likely to feel positive emotions such as happiness or enjoyment (Deaton and Arora, 2009). Mendelian randomisation studies have shown that benefits of taller body height could be direct/causal but also otherwise explained, with the effect remaining independent of body mass index (Tyrrell et al., 2016).

Previous studies on height in Switzerland based on various data sources have shown that residential region (ZIP code), socioeconomic background, and migration background are important determinants (Vinci et al., 2019, The Emerging Risk Factors Collaboration, 2012, Staub et al., 1992). Both men and women with primary education levels are significantly shorter than people with tertiary education. Regarding migration background, there is also a gradient between people born in Switzerland and people born outside of Switzerland and Europe. There are even regional differences in body height between the different language regions in Switzerland. Following the well-known North-South gradient across Europe, people from the Italian-speaking region in southern Switzerland are typically shorter than those from the German-speaking regions in northern Switzerland (Vinci et al., 2019). However, previous studies on the Swiss adult population were not large enough in sample size to test whether this plateau was reached among different subgroups of the Swiss population or did not provide information on health status (beyond mortality Rohrmann et al., 2017) alongside height.

We analysed for the first time the Swiss Health Surveys (SHS) regarding height trends of the Swiss adult population. (Volken et al., 2017) Therefore, the aim of the present study was to investigate whether different subgroups have reached a plateau in body height. In particular, we were interested in whether there were differences between different groups of migration background and levels of education. Moreover, we wanted to know whether taller individuals of the Swiss population considered themselves as healthier and/or their quality of life as better than shorter individuals.

## 2 Materials and methods

We used data from the Swiss Health Surveys (SHS). The SHS is a cross-sectional survey that has been conducted every-five years since 1992 by the Federal Statistical Office. The SHS is a telephone interview survey supplemented by a written follow-up questionnaire (all questionnaires are validated (Storni and Lieberherr, 2017, Schweizerisches Bundesamt für Statistik (BfS), 2017)). The representative samples are drawn by the Swiss Federal Statistical Office and cover the three predominant language/geographic regions of Switzerland (German-, French-, and Italian-speaking Switzerland). The study populations include all persons aged >=15 years living in private households, including foreign nationals. To correct for the sampling strategy and non-response, survey results were weighted including calibration factors that consider sampling strategy and sociodemographic (including age and education level, but not parent’s country of origin) and geographical characteristics. A more detailed and precise description of the data collection, recruitment procedure, participant rates, and sample weight strategy was published elsewhere (Storni and Lieberherr, 2017, Schweizerisches Bundesamt für Statistik (BfS), 2017).

For the present study, we focus on the most recent SHS from 2017 because this latest survey was the first to ask about migration background in more detail (beyond nationality). The earlier SHS surveys are only used for sensitivity analyses (SHS 1992, 1997, 2002, 2007, 2012). The original 2017 sample included n = 22,134 participants. We excluded n = 60 participants (0.27 %) who were smaller than 130 cm, n = 1 (>0.00 %) participant who was taller than 210 cm, n = 1,551 participants (7.0 %) who were younger than 20 years old, as well as n = 1,087 participants with missing information about the country of origin of the mother or the father (4.9 %). This remained in a sample of 19,435 of participants (87.8 % of the initial data), which representative of 6,239,235 people in Switzerland.

### 2.1 Variables

The main outcome variable in this study, body height, was asked during the telephone interviews and was specified in cm.

Regarding health status, we used the following outcome variables (all were asked during the telephone interview). Because various studies have shown a positive association between more back pain and greater height (Heuch et al., 2015, Hershkovich et al., 2013), we included the question “Did you have back or lower back pain in the last 4 weeks?” with the categorized answers “none”, “some”, and “strong”. Since there is a relatively large body of literature showing that there is a positive association between good cardiovascular health and greater height (Perkins et al., 2016, Bourgeois et al., 2017, Cochran et al., 2021, Islam et al., 2020, Nüesch et al., 2016, Lee et al., 2019, Henriksson et al., 2001), the following three questions or variables were included: a) “Has a doctor or other medical professional ever told you that your blood pressure is too high?” (answers “yes” or “no”), b) “Has a doctor or other medical professional ever told you that your cholesterol level is too high?” (answers “yes” or “no”), and body mass index (calculated from self-reported height and weight, categorized into WHO groups). To assess the general health status of the participants, we included the question “What is your general state of health?” with the prespecified answers categorized into “very good” and “good”. Due to sample sizes, we summarized the remaining three answer categories into one category, “fair/bad/very bad”. Finally, because there is also literature documenting a positive association between greater life satisfaction and greater height (Perkins et al., 2016, Deaton and Arora, 2009, Wyshak and Laks, 2014), we included the question “How do you assess your quality of life in general?” (the answers are again “very good”, “good”, and “fair/bad/very bad”).

The country of origin of the participants' parents was classified into six large groups of countries of origin, namely, in countries belonging to Central/Northern/Western Europe (which also includes participants with parents from Switzerland), Southern Europe, Eastern Europe (which also includes participants with parents from South-East Europe), South America, Africa, or Asia. For all other subgroups, the sample size was too small, so these countries of origin were combined into “Other.” If the two parents of a participant did not belong to the same group of countries of origin, the participant was also assigned to the “Other” group. The nationality of the participants was classified into Central/Northern/Western Europe, Southeast Europe, Southern Europe, Switzerland, and others. Education level was available as three groups: primary (no degree or with a compulsory school degree), secondary (completed high school or apprenticeship), and tertiary education (higher degree requesting a high school diploma). To account for regional differences, we included the variable “Language region” (German-, French-, and Italian-speaking regions of Switzerland). The MS (MS = mobilité spatiale) region variable was also available in the dataset, which was used to show the average height in 106 MS regions of Switzerland on a map. The 106 MS regions are defined by the Federal Statistical Office and used as an intermediate microregional level for scientific and regional policy purposes. Urbanity was available as a binary variable (“urban” vs “rural”). The years of birth of the participants were divided into ten-year groups.

### 2.2 Ethics

The SHS data fall under the Swiss Federal Data Protection Act. Data collection, processing, and storage are regulated by law (Ordinance of 30 June 1993 on the Conduct of Federal Statistical Surveys (SR 431.012.1) and Ordinance of 19 December 2008 on the Federal Population Census (SR 431.112.1)). All personal data were collected voluntarily with the knowledge of the participants. The individual data are publicly available and were provided by the Federal Statistical Office (FSO) in fully anonymized form based on contractual agreement. Due to the anonymization of the data, based on the Human Research Act (HRA), there was no obligation to obtain approval from an ethics committee.

### 2.3 Statistical methods

In the main analysis, only the SHS of 2017 was used. All analyses were carried out separately for men and women. The analysis was restricted to participants aged 20 years and older, because we assume, based on the literature, that most of the participants had more or less completed their physical growth by this age, which allows us to focus the analysis on full-grown adult individuals (Bogin, 1999).

We used general additive models (GAM) to assess the nonlinear associations between body height and year of birth. GAM models are an extension of generalized linear models obtained by allowing not only linear associations but also non-linear terms (Wood, 2017). The non-linear term (smooth term) is predicted using smooth functions and allows to assess if an association between two variables is not equal over the entire distribution (i.e. linear). In our models, we used a smooth term for the independent variable year of birth. First, we computed an unadjusted GAM model containing only the smooth term year of birth and compared it to an adjusted model that has the following additional linear terms: nationality, education, urbanicity, and language region. In addition, for sensitivity analyses, we compared the adjusted model of SHS 2017 with earlier years of the SHS. Second, we stratified the analysis according to the six migration backgrounds and performed the same adjusted GAM model. Third, we stratified the analysis according to the three educational groups and performed GAM models, adding the following linear terms for the smooth term: nationality, urbanicity, and language region. Since we were interested in the differences in body height and trends among the individual subgroups, we decided to stratify the groups and not to include interaction terms in our models.

Multinomial logistic regression was used to predict probabilities of self-rated health determinants (having back pain, hypertension, high cholesterol level, high BMI, health status, quality of life) in relation to body height.

The predictions of the probabilities are shown for median age, secondary education, Swiss nationality, German-speaking region, and urban urbanity.

To examine the influence of each independent variable on the self-rated health determinants, we removed each independent variable from the model at a time and calculated the AIC (Akaike's information criterion). Next, the difference between the AIC for the full model M and the model with omission of an independent variable k was calculated, i.e., ΔAIC_k_ = AIC_k_-AIC_M_. The larger ΔAIC_k_ is, the more important the variable is in the model.

The regional differences in the average height of men and women according to place of residence in Switzerland by MS region are displayed in choropleth maps. For that, the average weighted height, adjusted for nationality, education, urbanicity, and year of birth in every MS region, was calculated for the combined data of SHS 2012 and 2017. For better illustration, the calculated average height was divided into 6 quantiles and the 6 quantiles were shown on the maps (Grossenbacher, 2016).

All statistical analyses were performed using R Version 4.0.5. The R package “mgcv” (Wood, 2017) was used to calculate the generalised additive models, and the package “nnet” was used to perform the multinomial logistic regression models (Venables, 2002). We used ggplot2 (Wickham, 2010) to produce all figures.

## 3 Results

Table 1 shows the descriptive statistics of the 2017 SHS sample. Overall, male participants were by average 177.0 cm (SD = 7.1) tall, and female participants were 164.6 cm (SD = 6.5) tall. The vast majority (68 % to 80 %) of males and females had Swiss nationality, were living in an urban area, and were speaking German. Approximately 70 % of the participants had parents from Central/Northern/Western Europe and about 10 % from Southern Europe (the other countries of origin were less frequent). In males, tertiary education was more frequent than in women (43.2 % vs 30.7 %), whereas excess weight (BMI>=25.0 kg/m2) was less prevalent in women than in men (33.4 % vs 52.7 %). Approximately 8 % of males and females rated their quality of life as “fair/bad/very bad”, and approximately 15 % of males and females rated their general health status as “fair/bad/very bad”. In approximately 28 % of the participants (males and females), a doctor told them that their blood pressure was too high. Elevated cholesterol was slightly more frequent in males than in females (20.2 % vs 16.9 %), whereas “some” or “strong” back pain was more frequent in women than in men (48.7 % vs 37.7 %).

**Table 1:** Descriptive characteristics (absolute and relative frequencies) of the SHS 2017 participants, unweighted (on the left) and using sample weights (on the right).

	Unweighted Sample			Weighted Sample			Mean Body Height (SD)	
	Overall	Men	Women	Overall	Men	Women	Men	Women
	19,435	9117	10,318	6,239,235	3,078,159	3,161,076		
**Year of Birth**	N	N	N	%	%	%	cm	cm
1919–1929	185	67	118	1	0.7	1.2	172.3 (6.7)	159.9 (6.7)
1930–1939	1239	567	672	6.1	5.2	6.9	173.1 (6.4)	161.1 (6.0)
1940–1949	2637	1259	1378	11.7	11.4	12.1	174.7 (6.4)	162.5 (6.0)
1950–1959	3303	1514	1789	15.1	14.8	15.3	176.2 (6.9)	163.9 (6.4)
1960–1969	4019	1986	2033	19.2	20	18.4	177.6 (6.9)	164.9 (6.4)
1970–1979	3409	1588	1821	17.8	18	17.6	178.7 (7.0)	166.2 (6.5)
1980–1989	2730	1237	1493	17.9	18	17.8	178.9 (6.9)	166.2 (6.5)
1990–1997	1913	899	1014	11.3	11.9	10.8	179.4 (7.4)	165.9 (6.3)

**Nationality**	N	N	N	%	%	%	cm	cm
Swiss	15,535	7058	8477	77.4	75.1	79.7	177.5 (6.9)	164.8 (6.4)
Central/Northern/Western Europe	1090	556	534	7.7	8.3	7.1	180.0 (7.2)	166.7 (7.1)
Other	297	146	151	2.4	2.4	2.4	175.0 (8.2)	162.6 (6.7)
South-East Europe	988	502	486	4.6	4.9	4.4	178.0 (7.0)	165.6 (6.4)
Southern Europe	1525	855	670	7.8	9.3	6.4	175.3 (7.3)	161.7 (6.8)

**Education**	N	N	N	%	%	%	cm	cm
Primary	2729	980	1749	12.8	10.1	15.5	174.1 (7.1)	161.6 (6.7)
Secondary	9948	4337	5611	50.0	46.6	53.3	177.1 (7.2)	164.5 (6.2)
Tertiary	6706	3785	2921	36.9	43.2	30.7	178.6 (6.7)	166.6 (6.4)
Missing	52	15	37	0.3	0.2	0.4	–	–

**Urbanicity**	N	N	N	%	%	%	cm	cm
Rural	6175	2924	3251	26.1	26.6	25.7	177.2 (6.9)	164.5 (6.4)
Urban	13,260	6193	7067	73.9	73.4	73.4	177.5 (7.2)	164.8 (6.6)

**Language**	N	N	N	%	%	%	cm	cm
French	5111	2316	2795	24.6	24.0	25.2	177.2 (7.0)	164.1 (6.6)
German	12,532	5926	6606	68.6	68.8	68.5	177.8 (7.1)	165.1 (6.5)
Italian	1792	875	917	6.8	7.3	6.4	175.1 (7.3)	162.7 (7.1)

**Parents' Country of Origin**	N	N	N	%	%	%	cm	cm
Africa	306	158	148	1.9	2.1	1.7	176.3 (7.0)	165.0 (6.5)
Asia	257	123	134	1.7	1.8	1.7	173.2 (7.8)	160.1 (6.0)
Central/Northern/Western Europe	14,060	6466	7594	71.4	70.3	72.4	177.9 (7.0)	165.1 (6.5)
Eastern Europe	1114	524	590	5.6	5.5	5.8	178.7 (7.0)	165.7 (6.3)
South America	212	59	153	1.3	0.9	1.8	174.3 (8.0)	161.4 (6.5)
Southern Europe	2261	1227	1034	11.4	12.9	9.9	175.1 (7.0)	161.8 (6.7)
Other	1225	560	665	6.6	6.6	6.6	178.4 (7.4)	165.2 (6.3)

**BMI**	N	N	N	%	%	%	cm	cm
Below 18.5	559	60	499	2.8	0.7	4.9	177.6 (9.8)	166.1 (6.3)
18.5– 24.9	10,206	3999	6207	53.6	46.1	60.8	178.0 (7.0)	165.3 (6.4)
25.0–29.9	6250	3804	2446	31.5	40.1	23.2	177.2 (7.0)	163.7 (6.5)
Above 29.9	2282	1214	1068	11.4	12.6	10.2	176.3 (7.7)	162.5 (6.8)
Missing	138	40	98	0.7	0.4	0.9	–	–

**Hypertension**	N	N	N	%	%	%	cm	cm
Yes	5656	2894	2762	27.9	29.5	26.3	176.4 (7.0)	163.3 (6.8)
No	13,716	6185	7531	71.8	70.1	73.5	177.9 (7.1)	165.2 (6.4)
Missing	63	38	25	0.3	0.4	0.2	–	–

**Cholesterin**	N	N	N	%	%	%	cm	cm
Yes	3901	2084	1817	18.5	20.2	16.9	176.1 (6.8)	163.3 (6.4)
No	15,440	6982	8458	81.0	79.2	82.7	177.8 (7.1)	165.0 (6.6)
Missing	94	51	43	0.5	0.6	0.4	–	–

**Back Pain**	N	N	N	%	%	%	cm	cm
None	10,972	5671	5301	56.7	62.2	51.3	177.4 (7.1)	164.8 (6.4)
Some	6922	2890	4032	35.5	31.7	39.1	177.7 (7.2)	164.7 (6.7)
Strong	1534	554	980	7.8	6.0	9.6	176.9 (6.9)	164.1 (6.9)
Missing	7	2	5	0.0	0.0	0.0	–	–

**Self-rated Quality of Life**	N	N	N	%	%	%	cm	cm
Very Good	8738	4069	4669	45.1	45.0	45.2	178.1 (7.0)	165.5 (6.4)
Good	9029	4303	4726	46.4	46.8	46.0	177.0 (7.1)	164.3 (6.6)
Fair or Bad or Very Bad	1644	740	904	8.4	8.1	8.7	176.3 (7.2)	162.6 (6.9)
Missing	24	5	19	0.1	0.0	0.2	–	–

**Self-rated General Health Status**	N	N	N	%	%	%	cm	cm
Very Good	7652	3645	4007	40.5	41.8	39.1	178.3 (7.0)	165.5 (6.3)
Good	8695	4108	4587	44.3	44.2	44.4	177.2 (7.1)	164.6 (6.5)
Fair or Bad or Very Bad	3078	1359	1719	15.2	13.8	16.5	175.5 (7.0)	162.9 (7.0)
Missing	10	5	5	0.1	0.1	0.0	–	–

**Fig. 1:**
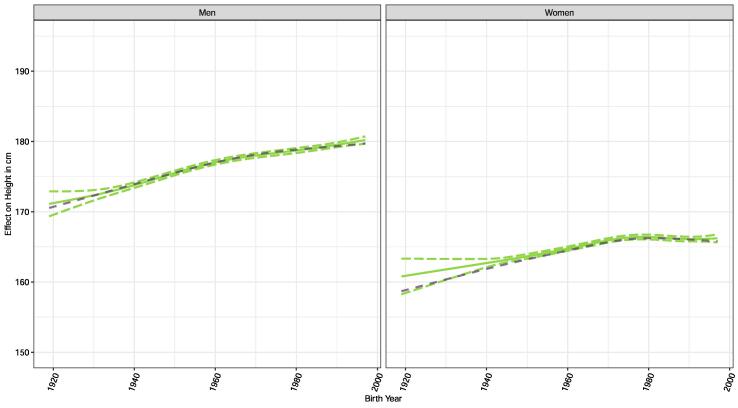
Unadjusted and adjusted temporal trends of average adult height across birth years among men (on the left) and women (on the right).

The means in Table 1 show that for men, the mean height increased by 7.1 cm from the oldest birth cohorts 1919–1929 (172.3 cm) to 179.4 cm for the youngest birth cohorts 1990–1997. For women, the respective increase in mean height was 6.0 cm (from 155.9 cm to 165.9 cm). A similarly large difference of 4.5 cm (men) and 5.0 cm (women) in mean height was found between participants with tertiary and primary education. Fig. 1 shows the modelled secular trend of height for men and women across birth years. Women reached a plateau in the 1970s, meaning that women born later were no longer significantly taller as adults than participants born before the 1970s. In men, this plateauing effect was less marked and there was more of a slowdown of the secular increase in adult height after the 1960s birth years. The unadjusted trends (dotted lines in Fig. 1) show good agreement and were slightly lower only for the early birth years up to 1940. Regression coefficients of the GAM crude and adjusted models are given in Supplement Table S2-3. When using a sensitivity analysis to compare the modelled trends from the 2017 SHS with equivalent models based on all earlier SHS (1992, 1997, 2002, 2007, 2012), we find very similar patterns and good agreement in terms of average height for men and women (Supplementary Fig. S1).

**Fig. 2:**
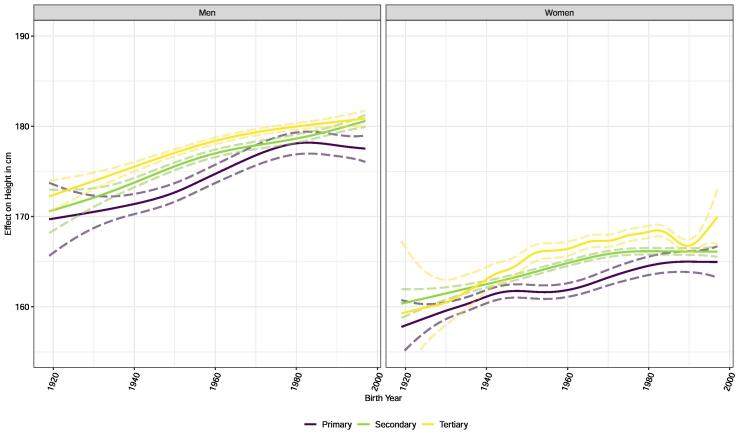
Adjusted temporal trends across education levels for men (on the left) and women (on the right).

If we look at the temporal trends by education level, we see that across the entire observation period, males and females with tertiary education were taller than participants with primary education (Fig. 2). For the small group of women with tertiary education, the most recent development is uncertain (as indicated by the wide confidence intervals). In most education subgroups, the slowdown in height increase can be seen from the 1970s birth years onwards. However, men with secondary education in particular seem to grow slightly taller again in the most recent birth years.

Looking at the time trends according to the countries of origin (Supplement Fig. S1), we see that women and men with parents from Central/Northern/Western Europe and from Eastern Europe were the tallest, whereas participants with parents from South America and Asia were the shortest. For participants with parents from Central/Northern/Western European, a plateau in average height was reached starting in the 1970s. For men with parents from Eastern Europe it was reached starting in the 1980s. Due to relatively small group sizes, the results for the remaining subgroups are not sufficiently clear.

The regional differences according to place of residence in Switzerland by MS region are shown in the maps in Supplementary Fig. S3. There is a large difference in average height between German-speaking northern Switzerland and Italian-speaking southern Switzerland. We can also see that the German-speaking subpopulation in the canton of Valais in southern Switzerland is among the tallest in Switzerland.

**Fig. 3:**
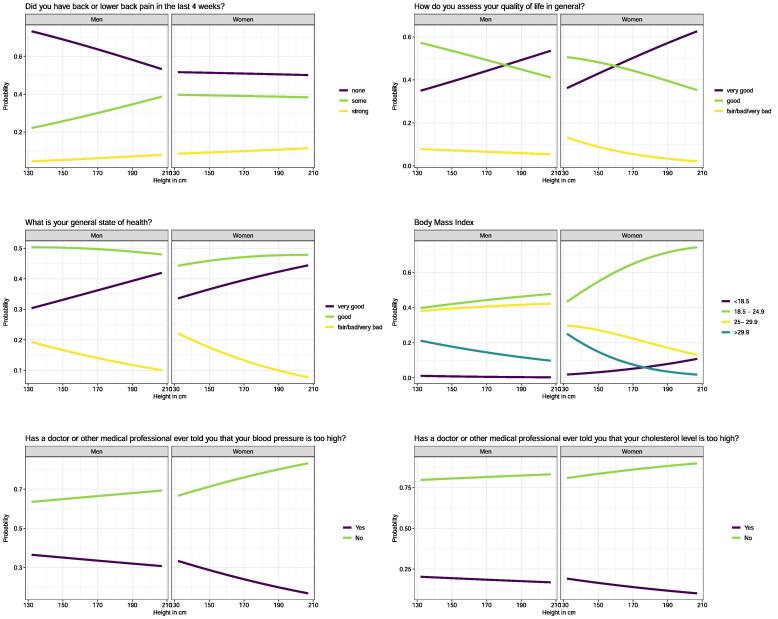
The probabilities of belonging to specific health outcome groups (lines) across the height spectrum (x-axis) for lower back pain, quality of life, general state of health, BMI, blood pressure, and cholesterol level. The probabilities are shown for median age, secondary education, Swiss nationality, German-speaking region, and urban urbanity.

The association between body height and self-declared aspects of health and life satisfaction is shown in Supplement Table S3-8. In Fig. 3, the probabilities of belonging to specific health outcome groups across the height spectrum are shown. In men, the probability of reporting lower back pain increased with increasing height, whereas this was not the case for women. The probability of reporting obesity decreased with increasing height in both sexes, but that pattern was more pronounced in women. With increasing height, men and women were less likely to report hypertension or high cholesterol levels. A marked pattern was found for the general state of health and the general quality of life: the taller the participants, the more likely the participants answered these questions with “very good”. The results of the ΔAIC examination (Supplement Table 2) revealed that body height was always an important cofactor to describe each of the self-rated health determinants.

## 4 Discussion

In this study, the largest and most recent representative dataset on body heights of adult women and men in Switzerland, the Swiss Health Survey (SHS) 2017, was analysed. We found that the increase in average body height slows down from around the 1970s birth cohorts. This is true for most subgroups of the population (educational level and partly for the different countries of origin). Women and men with tertiary education levels were taller than participants holding other education levels, and people from German-speaking northern Switzerland were taller than those in Italian-speaking southern Switzerland. Regarding countries of origin of the participant’s parents, which could be analysed in detail (and beyond only relying on nationality of the participants) for the first time in the SHS 2017, it can be seen that participants with parents from Central/Northern/Western Europe or Eastern Europe were taller than participants with parents from South America and Asia. We also found significant associations between body height and self-declared aspects of health and life satisfaction. Being taller was mostly an advantage (less overweight, better cardiovascular health, more life satisfaction, better overall health status), except for men having lower back pain.

Representative information on the body height of the adult population in Switzerland is still scarce. This study is based on the largest population-based survey dataset analysed to date. Previous studies analysed the menuCH survey 2013/2014 (n = approx. 2000) (Vinci et al., 2019) and the Swiss Household Panel 2004 (n = approx. 8000) (Kues, 2010). Regarding the trends in height development, we can confirm these earlier studies. The slowdown in the increase in height has also already been demonstrated at the national level on the basis of international consortium studies (Bentham et al., 2016). The reasons for the slowdown in height increase generally observed in northern and central Europe are not yet fully understood. The literature lists the following possible contributors. The most supported hypothesis is that in a now stable food and health environment, most people reach their full genetic growth potential (Marck et al., 2017). Following this argument, height would be a good indicator for deficiency but not for excess (Steckel, 2009). However, possible reasons also include aspects of dietary change, for example, when shares of consumed protein are stabilized, which are considered important in the context of physical growth (Vinci et al., 2019). In the SHS 2017, it was possible to determine the parents' countries of origin of the participants, and for certain subgroups of countries of origin, the sample size was large enough to solidly estimate subgroup trends. However, subgroups for other countries of origin were still too small to produce more precise trend estimates.

The increase in mean height at the beginning of the birth cohorts displayed by the Swiss Health Survey reflects a well-known phenomenon, the so-called secular trend. Since the 19th century, the average height of people in the Western world has steadily increased (along with faster physical maturation and earlier onset of menarche) (Bentham et al., 2016, Hatton and Bray, 2010, van Zanden et al., 2014). A complex set of environmental improvements in living conditions, nutrition, hygiene, the disease environment, greater health/socioeconomic equality and reduced physical stress are commonly cited as determinants of this so-called secular trend, along with other cofactors such as epigenetics, assortative mating or increased gene flow due to increased mobility (Stulp and Barrett, 2016, McEvoy and Visscher, 2009, Bogin, 1999). Although genetic influence is strong at the individual level (ca. 80 %), the steady increase in mean body height over the last 150 years has not been attributed to genetic changes. It is assumed that the genes responsible for growth could not change to such an extent in such a short period of time (Stulp and Barrett, 2016, McEvoy and Visscher, 2009, Wells and Stock, 2011, Bogin, 2013). Research on the secular increase in height over time has existed since the 20th century, with work gaining momentum during the 1970s and 1980s (Tanner et al., 1982, Poitevineau et al., 1977, Wolanski, 1984), and recently a large consortium has documented worldwide trends (Bentham et al., 2016).

With regard to regional differences in body height within Switzerland, we confirm the general and well-known North-South height gradient across Europe and thus also between the German-speaking north and the Italian-speaking south of Switzerland. The well documented difference in body height between northern and southern Europeans is explained by both interacting endogenous (due to genes) and exogenous (due to the environment) factors (McEvoy and Visscher, 2009). In addition to genetic differences (Novembre et al., 2008), biological factors (Bergmann's rule) and socio-economic aspects, different dietary patterns, for example with regard to dairy products, also play a role (McEvoy and Visscher, 2009). However, how well the body can process cow's milk products (lactose tolerance) is again genetically determined (Bogin, 1999). Our results also reveal fine-regional aspects that have already been shown in small-scale studies on Switzerland (Panczak et al., 2017): The present study confirmed that people from the small German-speaking area of the southern canton of Valais were tall compared to the French- and Italian-speaking areas in southern Switzerland (Panczak et al., 2017). It is possible that the documented genetic differences between the cultural language regions in Switzerland (Novembre et al., 2008) are also reflected in these local differences in mean body height in Southern Switzerland. However, in the area of environmental factors, which is less important than genetics with regard to height, aspects such as cultural dietary differences (Matthes et al., 2022) or restricted (also genetic) mobility due to alpine remoteness could also contribute.

Another result of our study also fits well into the literature: That within the same population people with higher educational level as a proxy for higher socio-economic background are taller. These differences were more pronounced in the past, but can still be described today (Panczak et al., 2017). These differences are not only related to the different living standards (Kues, 2010) of the parents, but also to other phenomena, such as assortative mating (Stulp et al., 2017), when taller women enter into partnerships with taller men and have children who are then taller themselves, which can lead to a pronunciation of the differences between the different socio-economic strata. Moreover, economists and sociologists have shown that – within the same population and all other factors being equal, such as age and education – taller people are happier (Deaton and Arora, 2009), earn more (Komlos et al., 2016) and have better career opportunities. The latter phenomenon is explained, among other things, by the fact that taller people are considered to be more assertive in job interviews. Such height benefits within the same population go back in part to childhood and selection processes at school (Thompson, 2022). These and other factors may also contribute to the observed height differences between educational levels.

We confirm earlier studies in documenting a robust association between adult height and health. Additionally, in Switzerland, based on record linkage between health studies and the mortality register, it was shown that there is an association between body height and (cause-specific) mortality (Rohrmann et al., 2017). Here, we add to that and show that on the basis of cross-sectional data, there are signals that point in the same direction: taller height is associated with better cardiovascular health (including excess weight) and better health status in general. Reasons for the association between taller height and better cardiovascular health include, among others, larger coronary vessel diameters, elevated insulin-like growth factors, slower heart rate, and/or greater lung capacity in taller people (Islam et al., 2020). Within the same population, shorter adult height is believed to reflect poor nutrition and/or lower socioeconomic circumstances in childhood, which was, to a certain degree controlled for – at least in adulthood – in our analysis (Collaboration, 2012). Due to the small number of SHS 2017 participants reporting cancer diagnoses in the interview, the association between cancer and height could not be assessed in our study. The importance of body height as a cofactor was medium-like in our models, indicating a certain relevance of this factor.

Health is certainly also one of the contributors to the association between height and life satisfaction found in our analysis. However, other longer-term aspects also play a role here, which we were unable to investigate in our analysis. It is hypothesized that within the same population a lack of (parental) investment in high-quality nutrition, hygiene, health, education, and positive environmental conditions in childhood leads to children not reaching their full physical development potential. This in turn, has a negative impact on health, emotional, reproductive, educational, and labour market outcomes later in life, which is mirrored in lower self-declared life satisfaction (Height, 2019).

Our study has strengths and weaknesses similar to those of many previous studies based on Swiss Health Surveys. One of the strengths is that our study is based on a comprehensive survey and a large representative sample of the general Swiss population. It is the largest representative study of body height in Switzerland to date on the adult population. In particular, the size of the dataset also allowed us to look at time trends by subgroup. Another strength is that in the 2017 SHS, the country of origin of the participants' parents was surveyed in more detail for the first time. This makes it possible to provide more precision here than was previously possible via information on the respondent's own nationality. However, the dataset, and thus our analysis, also has some important limitations. First, the data on body height and on health are based on self-declared data, which is known to be potentially biased. Especially in the case of self-reported body height, it is known that this tends to be overestimation and that this overestimation increases with age. However, it is known from studies comparing self-reported and measured body heights that the observed patterns do not differ significantly in time trend and subgroup differences. Second, large population-based health studies have tended to face declining participation rates in recent years. This trend has been halted in SHS since 2012 (the last study) and 2017 by incorporating alternative ways of contacting study participants. However, the possibility of a so-called healthy participant bias certainly remains if the healthier part of a population is more likely to participate in studies of this type than the unhealthier part. Third, SHSs are purely cross-sectional studies. The patterns shown are therefore only association, and no statements about causality can be made. Fourth, the health survey questionnaires do not allow an assessment of which childhood diseases affecting growth may have influenced adult height. Fifth, we could only control our analysis for those factors that were available as variables in the health surveys. Even though we think we have made a good selection from this existing variable catalogue, we cannot represent the whole spectrum of multifactorial factors influencing body height. This is especially true for the important genetic and epigenetic factors, as well as for hormonal and metabolic factors and life circumstances in general, especially during childhood (Perkins et al., 2016, Bogin, 1999). Furthermore, body proportions (leg length) may also play a role (Bogin and Varela-Silva, 2010).

## 5 Conclusion

In the public health field, body height tends to still be underestimated as a relevant cofactor for health. This concerns both the monitoring of living standards of subgroups and the association with health and life satisfaction. Although adult body height can no longer be influenced by prevention programs, it can be influenced in childhood and during human growth. In view of the future, it is worthwhile to address social inequality and strengthen healthy living conditions starting in childhood.

## CRediT authorship contribution statement

**Sarah-Maria Müller:** Conceptualization, Writing – original draft, Writing – review & editing. **Joël Floris:** Writing – review & editing. **Sabine Rohrmann:** Writing – review & editing. **Kaspar Staub:** Supervision, Conceptualization, Methodology, Writing – original draft, Writing – review & editing. **Katarina L Matthes:** Methodology, Software, Formal analysis, Visualization, Writing – original draft, Writing – review & editing.

## Declaration of Competing Interest

The authors declare that they have no known competing financial interests or personal relationships that could have appeared to influence the work reported in this paper.

## Data Availability

The authors do not have permission to share data.
