# Supplementary material for: Body height among adult male and female Swiss Health Survey participants in 2017: Trends by birth years and associations with self-reported health status and life satisfaction
Source: Prev Med Rep. 2022 Sep 12;29:101980. doi: 10.1016/j.pmedr.2022.101980 (PMC9502675; doi:10.1016/j.pmedr.2022.101980)
Supplement: Supplementary data 1 [file mmc1.pdf]

Supplement

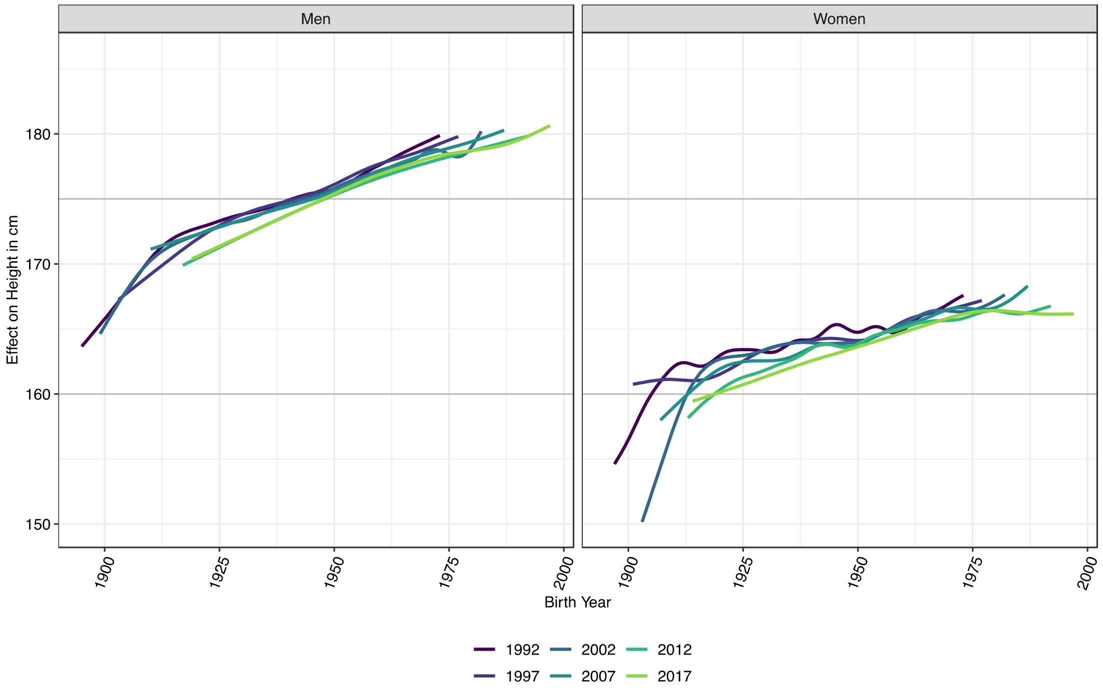

Supplementary Figure S1: Adjusted time trends for all six SHS since 1992, for men (on the left) and women (on the right).

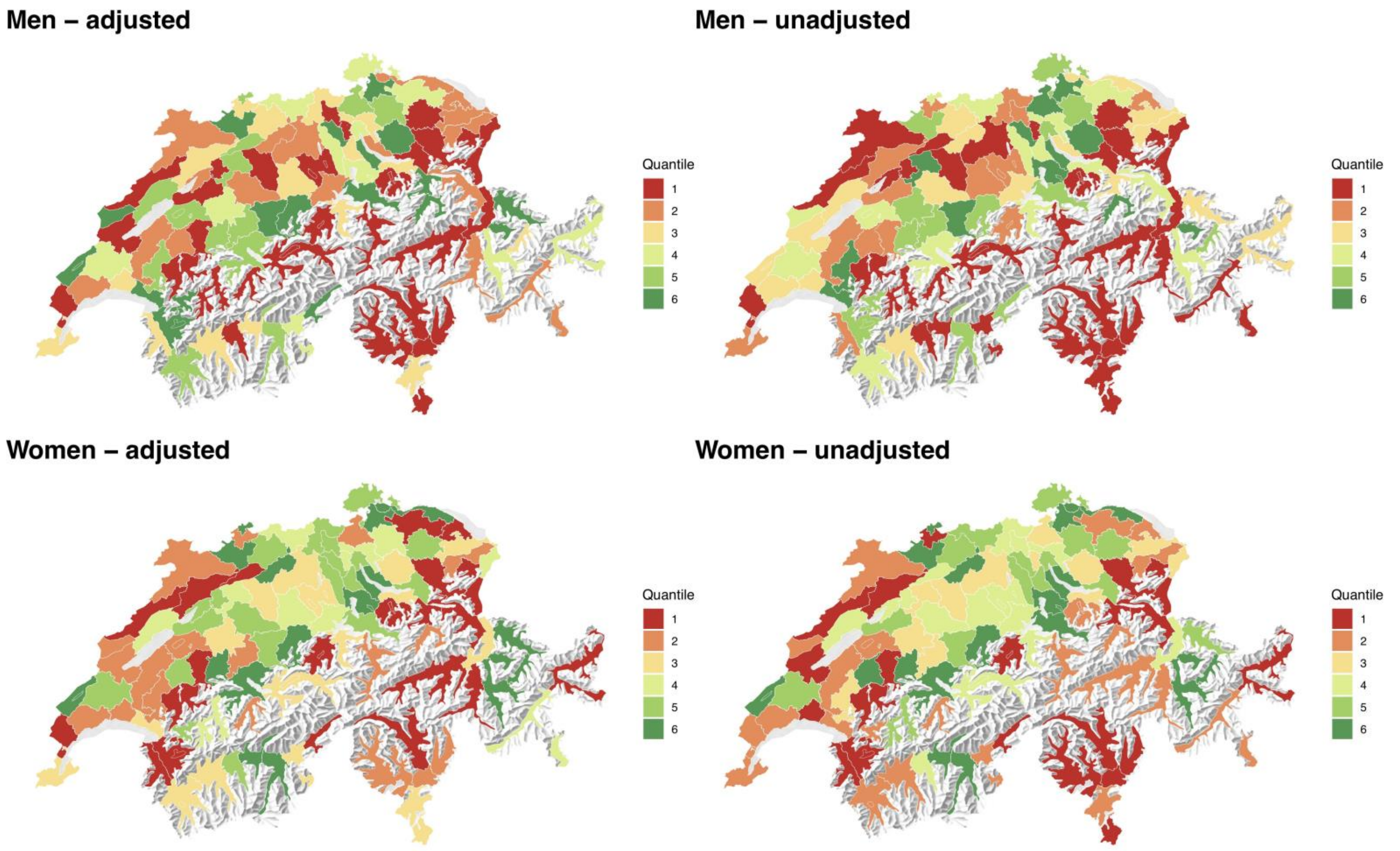

Supplementary Figure S2: Adjusted and unadjusted average height of men (upper map) and women (lower map) in the 2012 and 2017 SHS, displayed for the 106 MS-regions of Switzerland. The average height is shown in 6 quantiles.

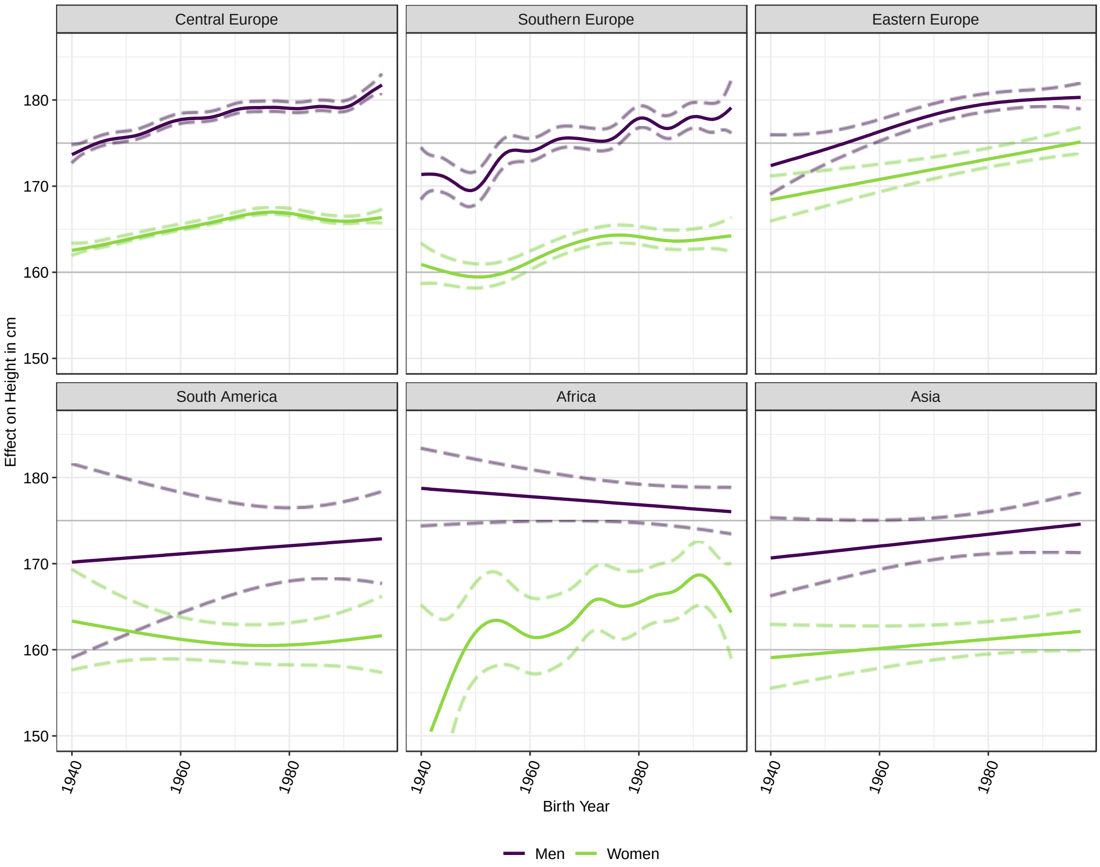

Supplementary Figure S3: Adjusted temporal trends across birth years for groups of countries of origin of participant's parents. Central Europe includes also Northern and Western Europe.

Men

**Back Pain**	Δ AIC	**BMI**	Δ AIC	**Health Status**	Δ AIC	**Quality of life**	Δ AIC	**Hypertension**	Δ AIC	**High cholesterol**	Δ AIC
Education	31928.7	Age	88917.0	Age	230429.6	Education	104706.2	Age	280977.6	Age	250829.8
Age	13547.3	Education	38689.3	Education	62565.0	LanguageRegion	47990.2	Education	8663.2	LanguageRegion	11841.7
Height	12083.3	Nationality	28365.4	Nationality	18767.4	Nationality	27377.3	Nationality	4958.9	Education	5275.1
Nationality	7882.0	Urbanity	3973.3	Height	9803.2	Height	9654.5	Height	4612.7	Height	4199.2
LanguageRegion	5184.0	LanguageRegion	2712.8	LanguageRegion	9351.4	Age	5020.2	LanguageRegion	1547.2	Nationality	3822.5
Urbanity	208.5	Height	2562.9	Urbanity	446.5	Urbanity	551.5	Urbanity	290.0	Urbanity	158.9

Women

**Back Pain**	Δ AIC	**BMI**	Δ AIC	**Health Status**	Δ AIC	**Quality of life**	Δ AIC	**Hypertension**	Δ AIC	**High cholesterol**	Δ AIC
Education	46413.3	Education	68495.6	Education	116450.3	Education	114892.3	Age	300605.5	Age	178816.3
Height	14339.3	Age	59743.4	Age	113222.7	LanguageRegion	60764.4	Education	31186.1	Education	9850.6
Age	7304.8	Height	29099.8	Height	19698.1	Nationality	31348.6	Height	9478.7	Height	7999.3
Nationality	6240.3	Nationality	13614.6	Nationality	14874.7	Height	23674.5	LanguageRegion	2605.0	LanguageRegion	2828.8
LanguageRegion	2812.6	Urbanity	3942.6	LanguageRegion	11877.4	Urbanity	3434.0	Nationality	1368.8	Nationality	2260.7
Urbanity	1123.2	LanguageRegion	1648.5	Urbanity	2929.6	Age	1462.4	Urbanity	321.2	Urbanity	2135.4

Supplement Table S1: ΔAIC (Akaike's information criterion) for consumption data including nationality and urbanity, the higher the ΔAIC is, the more important the variable.

Women	Estimate	SE	t.value	p.value
**Intercept**	163.29	0.3	497.0	<0.001
**Urbanicity**				
Rural*	-	-	-	-
Urban	0.17	0.1	1.1	0.254
**Nationality**				
Swiss*	-	-	-	-
Central/Northern/Western Europe	0.74	0.2	3.0	0.003
Other	-2.93	0.4	-7.0	<0.001
South-East Europe	-0.19	0.3	-0.6	0.542
Southern Europe	-2.56	0.3	-9.0	<0.001
**Language**				
German*	-	-	-	-
French	-0.80	0.2	-5.3	<0.001
Italien	-0.79	0.3	-2.8	0.005
**Education**				
Primary*	-	-	-	-
Secondary	1.68	0.2	8.3	<0.001
Tertiary	3.11	0.2	14.1	<0.001

* Reference

Men	Estimate	SE	t.value	p.value
**Intercept**	175.14	0.3	591.5	<0.001
**Urbanicity**				
Rural*	-	-	-	-
Urban	0.41	0.2	2.5	0.013
**Nationality**				
Swiss*	-	-	-	-
Central/Northern/Western Europe	1.68	0.3	6.3	<0.001
Other	-2.73	0.5	-5.6	<0.001
South-East Europe	-0.11	0.3	-0.3	0.749
Southern Europe	-1.39	0.3	-4.8	<0.001
**Language**				
German*	-	-	-	-
French	-0.44	0.2	-2.5	0.0131
Italien	-1.56	0.3	-5.0	<0.001
**Education**				
Primary*	-	-	-	-
Secondary	1.94	0.3	7.1	<0.001
Tertiary	3.27	0.3	11.5	<0.001

* Reference

Women	edf	F	p.value
**Unadjusted**			
s(Year of birth)	3.65	85.4	<0.001
**Adjusted**			
s(Year of birth)	4.53	53.4	<0.001

Men	edf	F	p.value
**Unadjusted**			
s(Year of birth)	2.82	122.7	<0.001
**Adjusted**			
s(Year of birth)	3.38	126.3	<0.001

Supplement Table S2: Regression coefficients of the crude and adjusted GAM models

Back pain

**Women**	**some vs none**	**strong vs none**
**Urbanicity**		
Rural*	-	-
Urban	0.944 (0.939 - 0.949)	1.096 (1.086 - 1.106)
**Nationality**		
Swiss*	-	-
Central/Northern/Western Europe	1.048 (1.039 - 1.058)	0.999 (0.982 - 1.016)
Other	0.721 (0.709 - 0.733)	1.153 (1.125 - 1.183)
South-East Europe	0.849 (0.839 - 0.859)	1.218 (1.196 - 1.241)
Southern Europe	1.197 (1.185 - 1.210)	1.510 (1.485 - 1.534)
**Language**		
German*	-	-
French	1.048 (1.042 - 1.054)	1.105 (1.095 - 1.116)
Italien	0.875 (0.865 - 0.884)	0.711 (0.699 - 0.724)
**Education**		
Primary*	-	-
Secondary	0.841 (0.835 - 0.847)	0.595 (0.589 - 0.602)
Tertiary	0.723 (0.717 - 0.729)	0.357 (0.352 - 0.362)
**Age**	0.999 (0.999 - 0.999)	1.010 (1.009 - 1.010)
**Height**	1.000 (1.000 - 1.000)	1.004 (1.004 - 1.004)

* Reference

**Men**	**some vs none**	**strong vs none**
**Urbanicity**		
Rural*	-	-
Urban	0.978 (0.973 - 0.984)	1.064 (1.052 - 1.076)
**Nationality**		
Swiss*	-	-
Central/Northern/Western Europe	1.099 (1.090 - 1.109)	1.217 (1.195 - 1.240)
Other	0.783 (0.769 - 0.797)	1.231 (1.195 - 1.267)
South-East Europe	1.052 (1.039 - 1.064)	2.087 (2.049 - 2.125)
Southern Europe	1.083 (1.073 - 1.094)	0.927 (0.910 - 0.945)
**Language**		
German*	-	-
French	1.228 (1.221 - 1.235)	1.193 (1.179 - 1.207)
Italien	1.085 (1.074 - 1.097)	1.245 (1.221 - 1.270)
**Education**		
Primary*	-	-
Secondary	1.048 (1.039 - 1.058)	0.640 (0.631 - 0.650)
Tertiary	0.905 (0.897 - 0.914)	0.338 (0.332 - 0.343)
**Age**	1.005 (1.005 - 1.005)	1.016 (1.016 - 1.017)
**Height**	1.012 (1.011 - 1.012)	1.012 (1.012 - 1.012)

* Reference

Supplement Table S3: Odds Ratios and 95% confidence intervals of multinomial regression analysis of self-rated health determinant: back pain

Quality of life Women	good vs very good	bad[1] vs very good
**Urbanicity**		
Rural*	-	-
Urban	1.095 (1.089 - 1.101)	1.340 (1.326 - 1.354)
**Nationality**		
Swiss*	-	-
Central/Northern/Western Europe	0.948 (0.940 - 0.957)	1.245 (1.223 - 1.268)
Other	1.692 (1.663 - 1.722)	2.697 (2.632 - 2.763)
South-East Europe	1.803 (1.781 - 1.825)	1.874 (1.835 - 1.914)
Southern Europe	1.954 (1.930 - 1.978)	2.778 (2.734 - 2.823)
**Language**		
German*	-	-
French	1.543 (1.534 - 1.552)	2.704 (2.678 - 2.731)
Italien	1.693 (1.673 - 1.712)	3.753 (3.694 - 3.813)
**Education**		
Primary*	-	-
Secondary	0.626 (0.621 - 0.631)	0.423 (0.418 - 0.427)
Tertiary	0.395 (0.391 - 0.398)	0.152 (0.150 - 0.154)
**Age**	1.003 (1.003 - 1.003)	1.003 (1.003 - 1.003)
**Height**	0.988 (0.988 - 0.988)	0.969 (0.969 - 0.969)

* Reference

[1] bad also includes fair and very bad

Quality of life Men	good vs very good	bad[1] vs very good
**Urbanicity**		
Rural*	-	-
Urban	0.950 (0.945 - 0.956)	1.046 (1.035 - 1.056)
**Nationality**		
Swiss*	-	-
Central/Northern/Western Europe	1.391 (1.379 - 1.403)	1.752 (1.724 - 1.782)
Other	0.872 (0.858 - 0.887)	1.516 (1.480 - 1.553)
South-East Europe	1.790 (1.769 - 1.812)	2.677 (2.628 - 2.727)
Southern Europe	1.481 (1.466 - 1.496)	1.869 (1.841 - 1.897)
**Language**		
German*	-	-
French	1.457 (1.449 - 1.466)	2.427 (2.402 - 2.452)
Italien	1.880 (1.859 - 1.902)	3.514 (3.457 - 3.572)
**Education**		
Primary*	-	-
Secondary	0.668 (0.662 - 0.675)	0.420 (0.414 - 0.425)
Tertiary	0.394 (0.390 - 0.398)	0.169 (0.166 - 0.171)
**Age**	1.004 (1.004 - 1.005)	1.008 (1.007 - 1.008)
**Height**	0.990 (0.990 - 0.990)	0.989 (0.989 - 0.990)

* Reference

Supplement Table S4: Odds Ratios and 95% confidence intervals of multinomial regression analysis of self-rated health determinant: quality of life

**General state of health**<br>**Women**	**good vs very good**	**bad[1] vs very good**
**Urbanicity**		
Rural*	-	-
Urban	1.068 (1.062 - 1.074)	1.249 (1.239 - 1.259)
**Nationality**		
Swiss*	-	-
Central/Northern/Western Europe	1.067 (1.057 - 1.078)	1.355 (1.336 - 1.375)
Other	1.951 (1.917 - 1.986)	2.018 (1.969 - 2.068)
South-East Europe	1.055 (1.042 - 1.068)	1.668 (1.640 - 1.696)
Southern Europe	1.235 (1.220 - 1.250)	1.766 (1.741 - 1.793)
**Language**		
German*	-	-
French	1.117 (1.110 - 1.123)	1.158 (1.149 - 1.168)
Italien	1.707 (1.686 - 1.728)	2.083 (2.052 - 2.114)
**Education**		
Primary*	-	-
Secondary	0.642 (0.636 - 0.648)	0.377 (0.374 - 0.381)
Tertiary	0.429 (0.425 - 0.433)	0.183 (0.181 - 0.185)
**Age**	1.015 (1.015 - 1.016)	1.036 (1.036 - 1.037)
**Height**	0.997 (0.997 - 0.998)	0.982 (0.982 - 0.983)

* Reference

[1] bad also includes fair and very bad

**General state of health**<br>**Men**	**good vs very good**	**bad[1] vs very good**
**Urbanicity**		
Rural*	-	-
Urban	1.034 (1.029 - 1.040)	1.094 (1.085 - 1.104)
**Nationality**		
Swiss*	-	-
Central/Northern/Western Europe	0.914 (0.906 - 0.922)	1.020 (1.006 - 1.035)
Other	0.654 (0.643 - 0.666)	1.033 (1.008 - 1.058)
South-East Europe	0.759 (0.750 - 0.768)	2.067 (2.035 - 2.099)
Southern Europe	1.184 (1.172 - 1.195)	1.229 (1.211 - 1.246)
**Language**		
German*	-	-
French	0.995 (0.989 - 1.001)	1.272 (1.260 - 1.283)
Italien	1.317 (1.303 - 1.332)	1.940 (1.911 - 1.969)
**Education**		
Primary*	-	-
Secondary	0.851 (0.843 - 0.860)	0.586 (0.579 - 0.593)
Tertiary	0.642 (0.636 - 0.648)	0.284 (0.281 - 0.288)
**Age**	1.024 (1.024 - 1.024)	1.055 (1.055 - 1.055)
**Height**	0.995 (0.995 - 0.995)	0.987 (0.987 - 0.987)

* Reference

Supplement Table S5: Odds Ratios and 95% confidence intervals of multinomial regression analysis of self-rated health determinant: general health status

**BMI**<br>**Women**	**18.5 - 24.9 vs <18.5**	**25 - 29.9 vs <18.5**	**>29.9 vs <18.5**
**Urbanicity**			
Rural*	-	-	-
Urban	0.957 (0.945 - 0.969)	0.894 (0.882 - 0.906)	0.733 (0.723 - 0.744)
**Nationality**			
Swiss*	-	-	-
Central/Northern/Western Europe	1.004 (0.984 - 1.024)	1.050 (1.028 - 1.073)	1.155 (1.127 - 1.184)
Other	0.607 (0.589 - 0.626)	1.148 (1.112 - 1.186)	1.270 (1.225 - 1.316)
South-East Europe	0.872 (0.851 - 0.893)	1.061 (1.034 - 1.090)	1.396 (1.356 - 1.436)
Southern Europe	1.109 (1.082 - 1.137)	1.537 (1.497 - 1.578)	1.928 (1.876 - 1.983)
**Language**			
German*	-	-	-
French	0.807 (0.798 - 0.817)	0.783 (0.773 - 0.793)	0.818 (0.807 - 0.830)
Italien	0.793 (0.775 - 0.811)	0.752 (0.734 - 0.770)	0.801 (0.780 - 0.822)
**Education**			
Primary*	-	-	-
Secondary	0.960 (0.943 - 0.978)	0.682 (0.669 - 0.695)	0.588 (0.576 - 0.599)
Tertiary	1.047 (1.027 - 1.067)	0.519 (0.508 - 0.529)	0.354 (0.346 - 0.362)
**Age**	1.006 (1.006 - 1.007)	1.025 (1.024 - 1.025)	1.023 (1.023 - 1.023)
**Height**	0.984 (0.984 - 0.984)	0.966 (0.966 - 0.967)	0.943 (0.943 - 0.944)

* Reference

**Men**	**18.5 - 24.9 vs <18.5**	**25 - 29.9 vs <18.5**	**>29.9 vs <18.5**
**Urbanicity**			
Rural*	-	-	-
Urban	0.711 (0.688 - 0.734)	0.667 (0.646 - 0.689)	0.559 (0.541 - 0.578)
**Nationality**			
Swiss*	-	-	-
Central/Northern/Western Europe	3.765 (3.741 - 3.789)	3.437 (3.414 - 3.460)	3.803 (3.769 - 3.838)
Other	7.877 (6.442 - 9.312)	7.474 (4.911 - 10.037)	6.224 (5.789 - 6.659)
South-East Europe	1.162 (1.152 - 1.171)	2.243 (2.226 - 2.260)	2.849 (2.821 - 2.876)
Southern Europe	1.077 (1.042 - 1.113)	1.518 (1.469 - 1.569)	1.877 (1.814 - 1.941)
**Language**			
German*	-	-	-
French	0.793 (0.770 - 0.816)	0.813 (0.789 - 0.837)	0.797 (0.774 - 0.822)
Italien	3.753 (3.716 - 3.791)	3.606 (3.570 - 3.642)	2.952 (2.917 - 2.987)
**Education**			
Primary*	-	-	-
Secondary	3.010 (2.942 - 3.079)	2.633 (2.574 - 2.694)	2.421 (2.365 - 2.479)
Tertiary	3.431 (3.355 - 3.508)	2.524 (2.469 - 2.581)	1.629 (1.592 - 1.667)
**Age**	1.042 (1.041 - 1.043)	1.062 (1.061 - 1.063)	1.065 (1.064 - 1.066)
**Height**	1.021 (1.020 - 1.021)	1.020 (1.019 - 1.020)	1.008 (1.007 - 1.008)

* Reference

Supplement Table S6: Odds Ratios and 95% confidence intervals of multinomial regression analysis of BMI.

Blood pressure

Women	yes vs no
**Urbanicity**	
Rural*	-
Urban	1.059 (1.052 - 1.066)
**Nationality**	
Swiss*	-
Central/Northern/Western Europe	1.115 (1.102 - 1.128)
Other	1.092 (1.070 - 1.115)
South-East Europe	1.268 (1.250 - 1.286)
Southern Europe	1.096 (1.082 - 1.110)
**Language**	
German*	-
French	0.889 (0.883 - 0.895)
Italien	0.765 (0.756 - 0.775)
**Education**	
Primary*	-
Secondary	0.696 (0.690 - 0.701)
Tertiary	0.530 (0.526 - 0.535)
**Age**	1.048 (1.048 - 1.048)
**Height**	0.988 (0.988 - 0.988)

* Reference

Men	yes vs no
**Urbanicity**	
Rural*	-
Urban	1.053 (1.047 - 1.060)
**Nationality**	
Swiss*	-
Central/Northern/Western Europe	0.752 (0.744 - 0.759)
Other	1.240 (1.218 - 1.262)
South-East Europe	0.959 (0.946 - 0.972)
Southern Europe	0.822 (0.813 - 0.831)
**Language**	
German*	-
French	0.982 (0.976 - 0.988)
Italien	0.792 (0.783 - 0.801)
**Education**	
Primary*	-
Secondary	1.111 (1.101 - 1.122)
Tertiary	0.943 (0.933 - 0.952)
**Age**	1.044 (1.044 - 1.044)
**Height**	0.997 (0.996 - 0.997)

* Reference

Supplement Table S7: Odds Ratios and 95% confidence intervals of multinomial regression analysis of self-rated health determinant: blood pressure

Cholesterol level

**Women**	**yes vs no**
**Urbanicity**	
Rural*	-
Urban	1.185 (1.177 - 1.194)
**Nationality**	
Swiss*	-
Central/Northern/Western Europe	1.278 (1.262 - 1.294)
Other	1.437 (1.406 - 1.469)
South-East Europe	1.036 (1.017 - 1.055)
Southern Europe	1.025 (1.010 - 1.040)
**Language**	
German*	-
French	1.202 (1.193 - 1.211)
Italien	1.216 (1.200 - 1.232)
**Education**	
Primary*	-
Secondary	0.907 (0.899 - 0.914)
Tertiary	0.868 (0.859 - 0.877)
**Age**	1.042 (1.042 - 1.042)
**Height**	0.990 (0.990 - 0.991)

* Reference

**Men**	**yes vs no**
**Urbanicity**	
Rural*	-
Urban	1.045 (1.038 - 1.052)
**Nationality**	
Swiss*	-
Central/Northern/Western Europe	0.815 (0.806 - 0.825)
Other	0.622 (0.606 - 0.639)
South-East Europe	1.187 (1.169 - 1.205)
Southern Europe	1.141 (1.128 - 1.154)
**Language**	
German*	-
French	1.443 (1.433 - 1.453)
Italien	1.485 (1.468 - 1.503)
**Education**	
Primary*	-
Secondary	1.107 (1.095 - 1.119)
Tertiary	1.064 (1.053 - 1.076)
**Age**	1.048 (1.047 - 1.048)
**Height**	0.997 (0.997 - 0.997)

* Reference

Supplement Table S8: Odds Ratios and 95% confidence intervals of multinomial regression analysis of self-rated health determinant: cholesterol level
